# Management of Osteoporosis in Parkinson's Disease: A Systematic Review of Clinical Practice Guidelines

**DOI:** 10.1002/mdc3.14311

**Published:** 2024-12-20

**Authors:** Mícheál Ó Breasail, Karan P. Singh, Fiona E. Lithander, Sze‐Ee Soh, Victor McConvey, Jennifer McGinley, Meg E. Morris, Peter R. Ebeling, Jesse Zanker, Ayse Zengin

**Affiliations:** ^1^ Department of Medicine, School of Clinical Sciences at Monash Health, Faculty of Medicine, Monash Medical Centre, Nursing and Health Sciences Monash University Clayton Victoria Australia; ^2^ Liggins Institute University of Auckland Auckland New Zealand; ^3^ Department of Physiotherapy and the Rehabilitation, Ageing and Independent Living (RAIL) Research Centre Monash University Melbourne Victoria Australia; ^4^ Fight Parkinson's Camberwell Melbourne Victoria Australia; ^5^ Department of Physiotherapy The University of Melbourne Parkville Victoria Australia; ^6^ Academic and Research Collaborative in Health (ARCH), and CERI La Trobe University Bundoora Victoria Australia; ^7^ Department of Medicine and Aged Care The Royal Melbourne Hospital, The University of Melbourne Parkville Victoria Australia

**Keywords:** Parkinson's disease, osteoporosis, fracture risk, clinical practice guidelines

## Abstract

**Background:**

Parkinson's disease (PD) is the fastest‐growing neurological disorder globally. Defining features include tremor, muscular rigidity, bradykinesia, and postural instability, which in combination with nonmotor symptoms such as cognitive impairment and orthostatic hypotension increase the risk of falls. Along with low bone mineral density, fracture risk is high in PD.

**Objectives:**

The aims were to identify and appraise clinical practice guidelines, consensus statements, and treatment algorithms containing recommendations for bone health in people with PD (PwP).

**Methods:**

We systematically searched 4 electroninc databases (MEDLINE, Embase, Emcare, and Web of Science) (n = 78), in addition to the websites of organizations, societies, and professional bodies focused on PD or osteoporosis (n = 28), up to April 22, 2024.

**Results:**

After duplicate removal, screening, and full‐text review, 6 records were included. Included records were appraised using the AGREE II (Appraisal of Guidelines for Research and Evaluation) tool. All records recognized bone health as a concern in PD, yet recommendations for fracture‐risk screening were inconsistent. Two of six records grouped PD under the broad category of neurological diseases. The acceptability and tolerance of anti‐osteoporosis medications in PwP was discussed only in 1 record, which incorporated national osteoporosis guidelines into a PD‐specific treatment algorithm.

**Conclusions:**

This review highlights that despite the documented high fracture rates of PwP, health professionals do not always have adequate resources to support them when considering how to manage osteoporosis. Osteoporosis screening and management needs to be incorporated into PD treatment guidelines, and equally providing specific recommendations for PwP related to bone health in national osteoporosis guidelines should be a priority given the high burden of fracture in the patient population.

Parkinson's disease (PD), a chronic progressive neurodegenerative disorder (NDD), is the fastest‐growing NDD globally^1^, ^2^, ^3^ and is second only to Alzheimer's disease in prevalence.^3^ Idiopathic PD is the most common form of parkinsonism with a prevalence of approximately 1% in those aged 65 to 69 years to nearly 3% in people aged 75 to 84 years.^4^ PD is characterized by a combination of tremor, rigidity, and bradykinesia, in addition to nonmotor symptoms^5^ that are also highly prevalent and heterogeneous,^6^ including orthostatic hypotension,^7^ cognitive impairment,^8^ muscle weakness/sarcopenia,^9^ and nutrition‐related issues.^10^ These exert a major impact on quality of life for people with PD (PwP) and their caregivers.^11^ Combinations of motor and nonmotor symptoms contribute to the high rate of falls in PwP,^12^ with approximately 60% of PwP falling each year and two thirds experiencing recurrent falls.^13^, ^14^, ^15^, ^16^, ^17^, ^18^, ^19^ Along with osteoporosis, this elevated fall risk is one of the major factors behind the high rate of fracture in PwP.

Osteoporosis is a highly prevalent metabolic bone disease; 1 in 3 women and 1 in 5 men over the age of 50 years will experience an osteoporotic fracture in their lifetime.^20^ Although primarily associated with aging, multiple pathologies, including a number of neurological conditions, are known to increase osteoporosis risk independent of age.^21^ Diagnosis is typically made using dual energy X‐ray absorptiometry (DXA)–assessed areal bone mineral density (aBMD), expressed as T‐scores which compare an individual's aBMD to that of a young healthy adult, at the total hip, femoral neck, and lumbar spine^22^, ^23^ or by the presence of a minimal trauma fracture. Pharmacological treatment options for osteoporosis include antiresorptive therapies (bisphosphonates and denosumab), and anabolic or mixed agents (romosozumab, abaloparatide, and teriparatide).^22^, ^23^ There are a number of lifestyle risk factors related to fracture risk that may be modifiable, which include fall reduction strategies, dietary modification, decreasing smoking and alcohol intake, maintaining optimal vitamin D status (≥50 nmol/L),^24^ and increasing physical activity and exercise, specifically those with resistance, balance, and weight‐bearing components.^25^, ^26^

PD is an acknowledged cause of secondary osteoporosis,^21^ but it remains unclear whether this or the high frequency of falls^27^ contributes more to the consistently high facture risk, particularly at the hip,^28^, ^29^, ^30^ seen in PD. The Global Longitudinal Study of Osteoporosis in Women found PD is the single‐greatest contributor to fracture in women.^31^ Similarly, in the United States, the osteoporotic fractures in men (MrOS) study reported that PwP had a lower aBMD, a greater number of fractures, and an increased mortality than men without PD.^32^, ^33^ Post fracture, PwP are more likely to develop complications,^34^, ^35^ have difficulty regaining mobility,^36^ and are twice as likely to die^37^ than those without PD. A recent systematic review and meta‐analysis of the risk of hip and nonvertebral fractures in parkinsonism (18 studies, n = 2,335,361) reported that PD increased the risk for both hip (2.40, 95% confidence interval [CI] 2.04–2.82) and nonvertebral fractures (1.80, 95% CI 1.60–2.01).^38^ Additionally, the relative risk for hip fracture was higher in men (2.93, 95% CI 2.05–4.18) than in women (1.81, 95% CI 1.61–2.04).^38^ Crucially, these authors highlighted the urgent need to reevaluate clinical guidelines on bone health in PwP to address this high risk of fracture. Data also suggest that risk of fracture in atypical parkinsonism may be higher than PD.^39^

Given the high fracture burden in PD, the aim of this study was to systematically review current guidelines for the management of bone health in PwP, to identify guideline adequacy for proactive bone health screening and management, as well as the gaps that may contribute to poorer bone screening and management in this population at high fracture risk.

## Patients and Methods

Using the Preferred Reporting Items for Systematic Reviews and Meta‐analysis (PRISMA) guidelines,^40^ a systematic review of 4 electronic databases (MEDLINE, Embase, Emcare, and Web of Science) was conducted up until April 22, 2024. This review was registered with PROSPERO (CRD42024539143). The full search strategy is included in Tables S1 and S2, but in brief, terms related to (1) PD were searched combined with (2) terms for clinical practice guidelines/treatment algorithms/position statements and (3) terms for bone health and osteoporosis. The search was limited to articles containing these terms in the title or abstract. No language or time restriction was applied at this stage. To complement the systematic search, the English‐language websites of PD/movement disorder societies, osteoporosis/bone health societies, and relevant professional bodies, and the Guidelines International Network (GIN) were searched. Only peer‐reviewed guidelines were considered for inclusion.

### Selection Criteria

Extracted records from the 4 databases were imported into Covidence (Veritas Health Innovation, Melbourne, Australia). After the removal of duplicate records, titles and abstracts were screened by 2 independent reviewers (F.E.L. and S.E.S.) and resolved by mutual agreement. Records were included for full‐text review if they appeared to be publications (eg, clinical practice guidelines, treatment algorithms, or expert/consensus statements) containing guidance for clinicians or allied health professionals on management, treatment, or prevention of osteoporosis/poor bone health in PD. Where a historic guideline was revised, only the most up‐to‐date version was considered for inclusion.

### Data Extraction and Quality Assessment

Descriptive details and recommendations were summarized and categorized by guideline recommendation focus (Table 1). The methodology used to form recommendations were extracted and categorized as evidence based, consensus based, or opinion based (or combinations of these). Two researchers (A.Z. and J.Z.) independently assessed the methodological quality of each included guideline using AGREE II (Appraisal of Guidelines for Research and Evaluation).^41^ Three or more discrepancies were resolved by mutual agreement. Quality scores were calculated for each of the 6 AGREE II domains, namely (1) scope and purpose, (2) stakeholder involvement, (3) rigor of development, (4) clarity of presentation, (5) applicability, and (6) editorial independence.^41^ Subsequently, domain scores were calculated by summing the scores of the individual items within a domain and expressing this as a percentage of the maximum possible score for a given domain.^41^ Based on AGREE II cutoffs these were then classed “high,” “moderate,” or “low” quality based on the research context: scores over 80% in ≥5 domains were classed as high quality, those greater than 60% in ≥4 domains as medium, and those below 59% as low quality.^41^ Summarized assessment results were then tabulated (Table 2).

**TABLE 1:** Summary of included guidelines

Details	Recommendation(s)	Methodology
van der Marck et al.,^46^ international “Consensus‐based clinical practice recommendations for the examination and management of falls in patients with Parkinson's disease”	Advice on generic and disease‐specific risk factors for falls in Parkinson's disease. Osteoporosis defined by authors as generic risk rather than PD‐specific risk for falls. “Method of ascertainment: History of prior fractures; Screen for risk factors for osteoporosis; Examine spine (pain, shape); Measure bone density.” “Who should be involved: General practitioner; Geriatrician; Clinical pharmacist.” “Suggested intervention: Anti‐osteoporotic treatment; Promote physical activity and exercise.”	Systematic search supplemented with additional references by the panel members, generic guidelines, PD‐specific guidelines and expert opinion.
NICE,^47^ England “NG71 Parkinson's disease in adults” Note: last updated May 2022	“Nutrition: 1.7.13 Advise people with Parkinson's disease to take a vitamin D supplement. See the NICE guideline on vitamin D for recommendations on vitamin D testing, and the NICE guidelines on falls in older people and osteoporosis. [2017]”	Evidence‐based: systematic review
Henderson et al.,^42^ England “Management of fracture risk in Parkinson's: A revised algorithm and focused review of treatments” Update of Lyell et al.^45^	Four‐step algorithm for the assessment and management of fracture risk in Parkinson's Step 1: Optimize vitamin D and calcium intake. Address lifestyle factors: alcohol, smoking, and physical activity. Step 2: Assess falls, previous fractures and back pain. Include spine imaging if occult vertebral fracture suspected. Step 3: Using FRAX, calculate the risk of MOF and hip fracture. Include PD as a secondary cause of secondary osteoporosis. For comorbid or life‐limited patients consider Qfracture. Step 4: Use the NOGG treatment algorithm (as weblink). Further detailed guidance provided on UK‐specific treatment options, indications for DXA referral, and additional considerations where treatment/assessment may be prioritized (ie, ≥2 falls in previous year) or facture risk should be inflated (ie, “10 yr hip fracture probability may be inflated by a factor of 30% per fall” up to 5 falls).	Expert opinion integrated with national evidence‐based best practice guidelines
SIGN,^44^ Scotland “SIGN142 Management of osteoporosis and the prevention of fragility fractures” First published March 2015 Revised June 2020 Revised January 2021	“People over the age of 50 with neurological disease (including Alzheimer's disease, Parkinson's disease, multiple sclerosis and stroke) may be considered for fracture‐risk assessment, particularly in the presence of other risk factors.”	Evidence based: systematic review
NOGG 2021, United Kingdom “Clinical guideline for the prevention and treatment of osteoporosis”	**Assessment of clinical risk factors:** “Several other drugs have been associated with increased fracture risk including antidepressants, antiparkinsonian drugs, antipsychotic drugs, anxiolytic drugs, benzodiazepines, sedatives, H2 receptor antagonists and proton pump inhibitors. The extent to which fracture risk is mediated by low BMD, falls risk or other factors, or indeed is definitely causal in each case, is not known.” **Clinical risk factors for osteoporosis/fractures, not accommodated in FRAX, which should trigger fracture‐risk assessment:** “Neurological/psychiatric disease e.g., Parkinson's disease and associated syndromes, multiple sclerosis, epilepsy, stroke, depression, dementia.”	Evidence based: systematic review
Carrol et al.,^43^ England “Addressing Comorbidities in People with Parkinson's Disease: Considerations from an Expert Panel”	**Recommendations to improve bone health in PwP:** “Following national guidelines for bone protection in PD in line with the recommendation from the revised algorithm BONE‐PARK, which applies The National Osteoporosis Guideline Group (NOGG) guidance specifically to PwP.” **Recommends clinician should consider:** “Providing patient with education and lifestyle advice to maintain their bone health; ° Reviewing medication: e.g., paying attention to management of orthostatic hypotension and the anticholinergic burden (prolonged exposure to anticholinergic drugs has been associated with an increased fall risk in older patients)^10^, ^28^, ^29^; ° Considering different types of physiotherapy^30^; Ensuring vitamin D replete, treating where deficiencies exist^22^, ^23^ supplementation to prevent deficiency when appropriate; Considering bisphosphonate therapy where appropriate.^22^, ^23^	Pharmaceutical‐funded roundtable meeting of a group of 10 experts to discuss recent PD developments with the purpose of developing expert advice to enhance clinical practice in PD.

Abbreviations: PD, Parkinson's disease; NICE, National Institute for Healthcare and Excellence; MOF, major osteoporotic fracture; DXA, dual energy X‐ray absorptiometry; NOGG, National Osteoporosis Guideline Group; SIGN, Scottish Intercollegiate Guidelines Network; BMD, areal bone mineral density; PwP, people with Parkinson's disease, FRAX.

**TABLE 2:** AGREE II scores for included guidelines

AGREE II domains	van der Marck et al.^46^	NICE^47^	Henderson et al.^42^	SIGN^44^	NOGG 2021	Carrol et al.^43^
1. Scope and purpose	95	98	98	100	100	90
2. Stakeholder involvement	67	98	43	93	98	43
3. Rigor of development	54	81	49	89	87	33
4. Clarity of presentation	64	100	98	100	100	55
5. Applicability	41	80	88	84	77	23
6. Editorial independence	79	60	96	57	100	61
Mean score	67	86	79	87	94	51
Overall quality	**Moderate**	*High*	**Moderate**	*High*	*High*	***Low***

*Note*: Domain scores were calculated according to the equation in the AGREE II manual. Mean overall scores 80% or more were classed as high quality (italic font), those between 60% and 79% as moderate (bold font), and those below 59% as low quality (bold‐italic font).

Abbreviations: AGREE, Appraisal of Guidelines for Research and Evaluation; NICE, National Institute for Healthcare and Excellence; SIGN, Scottish Intercollegiate Guidelines Network; NOGG, National Osteoporosis Guideline Group.

## Results

**FIG. 1:**
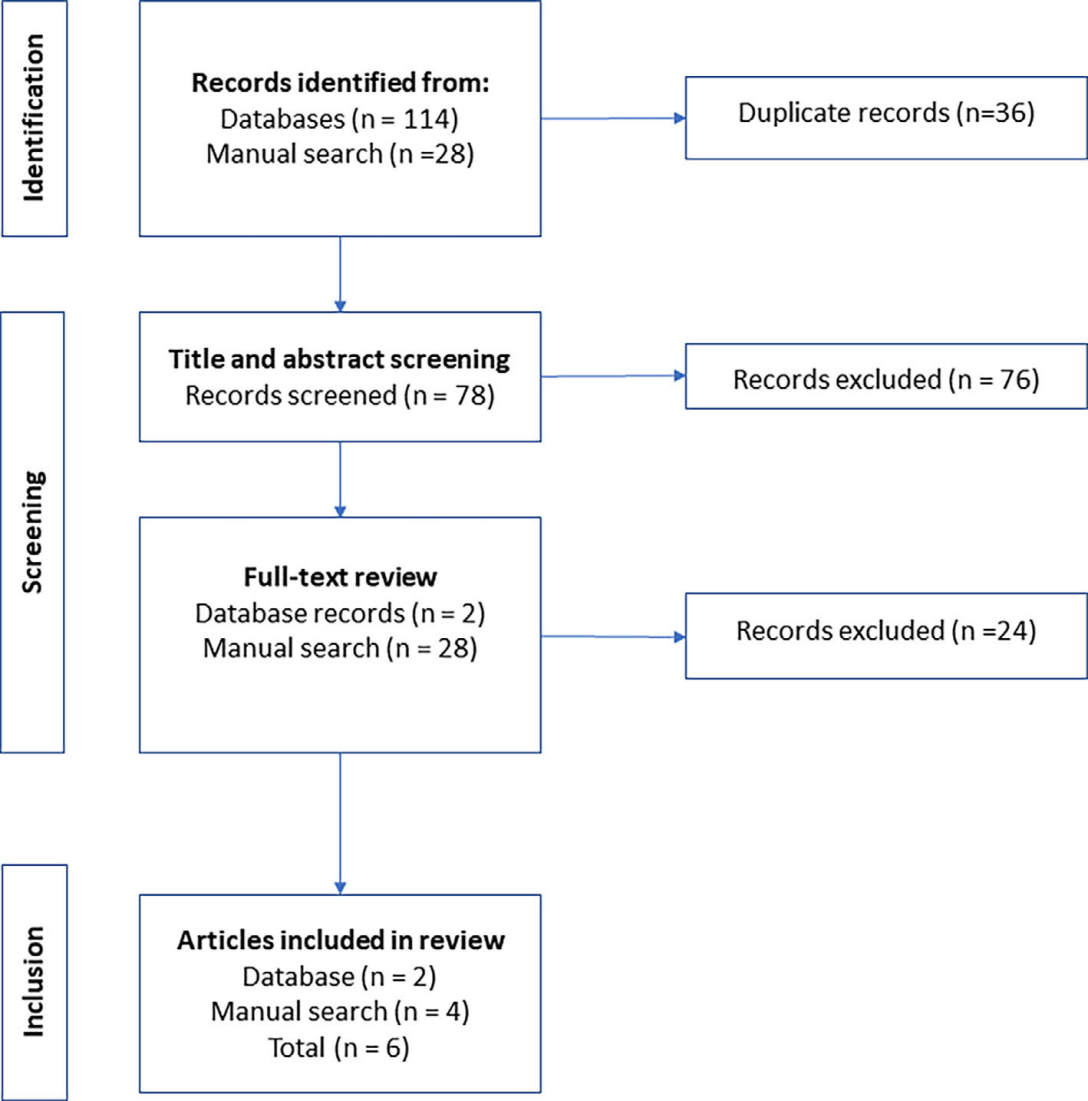
PRISMA (Preferred Reporting Items for Systematic Reviews and Meta‐analysis) flow diagram.

One hundred and fourteen were returned from database searches, with a further 28 records identified through manual searches (Fig. 1). After the removal of duplicates (n = 36), 78 titles and abstracts were screened, resulting in 2 records being selected for full‐text review. Four additional potential guidelines were identified after the full‐text review of 28 records identified from searching the GIN in addition to the English‐language websites of organizations and professional bodies whose work encompasses either bone health/osteoporosis or movement disorders/PD. This resulted in 6 guidelines being included in this review (Fig. 1).

### Details of the Records Included

The majority of included guidelines (5 of 6) originated from the United Kingdom.^22^, ^42^, ^43^, ^44^ These included clinical practice guidelines by the National Institute for Health and Care Excellence (NICE) in England NG71 “Parkinson's disease in adults,” the Scottish Intercollegiate Guidelines Network (SIGN) 142 “Management of osteoporosis and the prevention of fragility fractures,” and the 2021 NICE‐accredited National Osteoporosis Guideline Group (NOGG) guidelines. The remaining 3 records that were published in academic journals were a 2019 update to a 2014 treatment algorithm for the management of fracture risk in PD^42^, ^45^ a 2024 expert panel review addressing comorbidities in PD,^43^ and 2014 clinical practice recommendations for fall management In PD.^46^

### AGREE II: Quality of Guidelines

The quality of the included guidelines varied from low to high (Table 2). Based on the average scores of the 6 AGREE II domains, guidelines produced by SIGN142 (2021), NICE NG71 (2017), and NOGG (2021) were assessed to be of overall high quality (≥ 80%), Henderson et al. (2019) and van der Marck et al. (2014) of moderate quality (60%–79%), and Carroll et al. of low overall quality (<59%). All guidelines scored highly for the first domain of “scope and purpose.” High scores in domain 2 “stakeholder involvement” were obtained only by the SIGN142, NICE NG71, and NOGG (2021), guidelines which explicitly involved doctors, nurses, allied health professionals, and patients in guideline development. Similarly, only these guidelines scored highly under domain 3 “rigor of development,” and they were underpinned by systematic literature reviews or performed grading of the evidence that was included (Table 2). Four of the 6 guidelines were scored highly for “clarity of presentation,” which is based on recommendations being specific and unambiguous, with different options for disease management outlined and key recommendations being easily identifiable (Table 2). The editorial independence of all guidelines was difficult to assess as not all guidelines clearly provided the conflicts of interest of all involved in their development; only 2 of the 6 scored *high*.

### PD and Bone Health

Both NOGG and SIGN142 osteoporosis guidelines highlighted Parkinson's among other neurological diseases as clinical risk factors that “should” (NOGG) or “may” (SIGN) trigger fracture‐risk assessment (Table 1). NOGG also includes under the “Assessment of Clinical Risk Factors” a number of drug classes that may increase fracture risk. Many of these drugs are commonly used by PwP to manage motor and nonmotor symptoms, including antidepressants, antiparkinsonian drugs, antipsychotic drugs, anxiolytic drugs, and benzodiazepines (Table 1). No PD‐specific guidance relating to the use of fracture‐risk calculators or differences in managing osteoporosis in this patient group is provided. NICE NG71 recommends that clinicians advise PwP to take a vitamin D supplement and signposted to other NICE guidelines on vitamin D for recommendations on vitamin D testing, and the NICE guidelines on falls in older people and osteoporosis (Table 1). van der Marck et al. placed osteoporosis under generic risk factors for falls in PD providing summary advice regarding the “method of ascertainment” of osteoporosis (including “BMD assessment”), “who should be involved” (general practitioner, geriatrician, clinical pharmacist), and “suggested intervention” (anti‐osteoporotic treatment, physical activity).^46^ The BONE‐PARK algorithm was the only guideline identified that provided treatment options available, with particular consideration given to PD‐specific factors. Guidance is summarized in 4 steps, which focus on (1) vitamin D and calcium adequacy in addition to addressing lifestyle factors; (2) assessing falls, previous fracture, and back pain; (3) using FRAX, with PD considered a cause of secondary osteoporosis to calculate the risk of major osteoporotic fracture (MOF) and hip fracture (or Qfracture if otherwise indicated); and (4) applying the NOGG 2017 treatment algorithm (Table 1). However, due to the recent NOGG update,^22^ further iterative revision is now required to ensure advice is up to date and in line with national best practice. Carroll et al. advocated BONE‐PARK use in PwP and that clinicians should consider other factors amenable to intervention (Table 1).^43^ The potential use of menopause hormone therapy in preserving bone during the menopause transition was not raised in the context of Parkinson's in any of the guidelines.

### Nutrition and Bone Health in PD

Ensuring adequate serum vitamin D was highlighted in 3 guidelines.^42^, ^43^, ^47^ NICE NG71 recommended clinicians to advise PwP to take a vitamin D supplement and direct clinicians to the guidelines containing general populations guidance on vitamin D (PH56),^48^ falls in older people (CG161),^49^ and osteoporosis (CG146).^50^ Concern about the use of calcium supplements was noted through a claim of “the potential for higher adverse events, such as cardiovascular disease without any evidence of additional benefit.”^47^ Henderson et al. suggested recurrent falls among PwP support consideration of testing serum vitamin D.^42^ Calcium intake, alongside serum vitamin D sufficiency, was mentioned in 2 guidelines^42^, ^43^ but supplementation was explicitly recommended only in BONE‐PARK if dietary calcium intake was insufficient to meet requirements (Table 1). No guidelines mentioned the intake of protein or other macro‐ or micronutrients in the context of bone health.

### Physical Activity, Balance Training, Exercise, and Bone Health in PD

The importance of promoting physical activity and exercise among other lifestyle factors to maintain bone health in PD was acknowledged by 3 of the identified guidelines.^42^, ^43^, ^46^ NICE NG71 synthesized the evidence across a range of interventions related to physiotherapy and physical activity in PD, with outcomes including freezing of gait, falls, balance, and speed of gait.^47^ Although bone health or fractures were not included as outcomes, the physiotherapy‐focused recommendations in NICE NG71 likely have value from a musculoskeletal perspective through mechanisms of improved balance, physical function, and fall reduction. No guidelines promoted osteogenic exercise regimens (eg, resistance training) nor were the differences in the guidelines between load‐bearing and nonload‐bearing exercises.

In this systematic review of international clinical practice guidelines, we found markedly limited guidance for health professionals who care for PwP in the screening, treatment, and management of osteoporosis despite well‐documented high fracture rates in PD and associated morbidity and mortality.^36^ Neurology‐focused guidelines acknowledge bone health as an issue but refer only to general population osteoporosis guidance. Similarly, osteoporosis guidelines acknowledge PD as a cause of secondary osteoporosis and/or as a risk factor for falls.^51^, ^52^, ^53^, ^54^ Only 2 of these recommend that clinicians consider screening PwP to assess their fracture risk though this was generalized to cover several neurological diseases, for instance, multiple sclerosis. A single comprehensive treatment algorithm, BONE‐PARK, contained PD‐specific bone health recommendations integrated with national osteoporosis guidelines.^42^, ^45^ Several areas key to bone health, namely (1) fracture‐risk assessment, (2) osteoporosis medications, (3) bone‐specific nutrition advice, and (4) physical activity/exercise, were dealt with in an inconsistent manner in the literature.

### Appropriate Assessment of Fracture Risk

A key component of osteoporosis management is predicated on the identification of fracture risk through screening for fracture risk. A number of widely used fracture‐risk algorithms exist,^55^ with varying discriminatory power for fracture prediction.^56^, ^57^ Internationally, FRAX is used most widely and generates probabilities of 10‐year MOF in the general population based on clinical risk factors.^58^ However, neither PD nor falls are available as clinical risk factors in FRAX. Although the former can be pragmatically mitigated by selecting “secondary osteoporosis,” the fracture burden in PD exceeds other common causes of secondary osteoporosis such as chronic obstructive pulmonary disease, type 1 diabetes, and multiple sclerosis.^31^ This is supported by recent data, which suggest that FRAX underestimates MOF probability in people with Parkinson's.^59^ Launched in 2023, FRAXplus considers additional risk factors, for instance, falls in the past year; though to date, probabilities between FRAX and FRAXplus have not been compared in PwP. QFracture,^60^ which is based on UK data, estimates the 1‐ to 10‐year cumulative incidence of hip and/or MOF, and includes Parkinson's and falls as clinical risk factors. This ability to produce MOF probabilities <10 years is why Qfracture has been suggested for use in those with limited life expectancy or atypical parkinsonisms.^39^ Of the guidelines reviewed in the current systematic review, 3 explicitly recommended that PwP should have a fracture‐risk assessment^22^, ^42^, ^43^; SIGN suggests that they “may be considered for fracture‐risk assessment,” whereas van der Marck et al. refer to screening for osteoporosis risk factors and BMD assessment^46^ in limited detail.

**FIG. 2:**
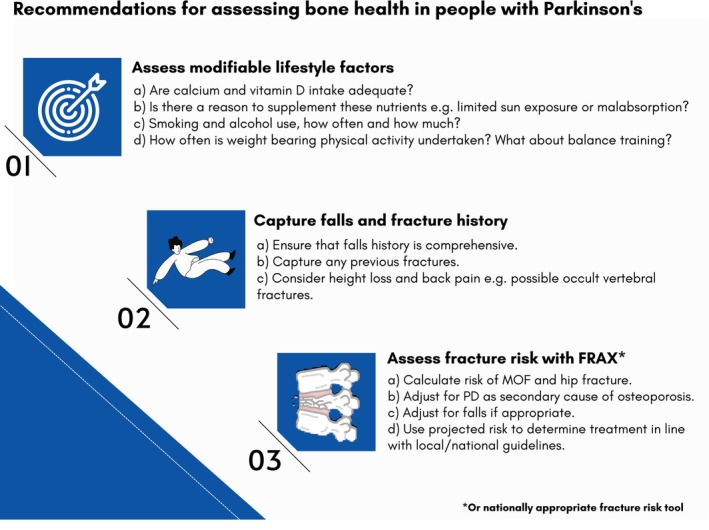
Recommendations for future guidelines.

Figure 2 summarizes the afore considerations that were absent from 1 or more of the included guidelines into a simple set of recommendations, which could be readily adapted in line with local or national osteoporosis guidelines.

### Treatment of Osteoporosis in PD

A variety of treatments exist for osteoporosis,^61^, ^62^ though PD adds additional complexity and potential barriers when prescribing anti‐osteoporosis medications due to common nonmotor symptoms such as dysphagia, slow gut motility, malabsorption, and cognitive impairment in addition to an already‐complex medication regimen. Oral bisphosphonates such as alendronate, ibandronate, and risedronate, which inhibit bone resorption, are generally the first line of treatment.^63^ However, daily bisphosphonates may be difficult to adhere to due to dysphagia and a strict dosing schedule (ie, early morning on an empty stomach, ideally before taking other medications, with a full glass of water and further maintenance of an upright position while fasting for ≥30 minutes post‐dose).^61^ Although liquid formulations (eg, alendronate)^64^ and monthly tablets (eg, ibandronate) are options, efficacy data are limited.^65^ Intravenous zoledronic acid (a bisphosphonate), typically received annually for 3 years, may be a particularly attractive alternative, with substantial fracture‐risk reductions reported in non‐parkinsonian cohorts (eg, 40% nonvertebral and 75% vertebral fracture relative risk reduction by 3 years).^66^ The efficacy of zoledronic acid in PwP is currently being assessed in the ongoing Trial of Parkinson's And Zoledronic Acid.^67^ Denosumab, a human monoclonal antibody to receptor activator of nuclear factor kappa‐Β ligand, may also be an attractive option in Parkinson's as it is administered subcutaneously on a 6‐monthly basis. However, the risk of secondary hypocalcemia is elevated in patients with poor renal function, and denosumab cessation can be associated with increased bone turnover and can increase the risk of vertebral fracture.^68^ Whereas both SIGN and NOGG guidelines contained detailed information on the indications and contraindications for anti‐osteoporosis medications for Scotland and England, respectively, BONE‐PARK explicitly presented recommendations based on (previous NOGG) guidelines considering the applicability and acceptability to PwP. Given that availability, pricing, and prescribing practices vary internationally, universal recommendations regarding anti‐osteoporosis prescribing cannot be made. However, there would be substantial benefit to PwP if clinicians were to follow the example of Henderson and colleagues with both iterations of the BONE‐PARK algorithm.^42^, ^45^

### Nutrition

Adequate nutritional intake to meet macro‐ (eg, protein) and micronutrient (ie, calcium and vitamin D) requirements is an important factor in maintaining bone health. This is of particular importance in PD where nutrition‐related issues are well documented,^10^ though often underappreciated.^69^ Although most of the guidelines assessed referred to ensuring serum vitamin D sufficiency via supplementation, little attention was given to the route of administration. Given the difficulty many PwP have with swallowing, it would be prudent to provide clinicians explicit guidance on the potential benefits and risks of bolus dosing with weekly or monthly oral dosing schedules. In contrast to vitamin D, advice regarding calcium intake/supplementation was lacking, and where provided, there was conflict between guidelines. NICE NG71 cautioned against the provision of calcium supplements citing concerns over cardiovascular health, whereas in line with the current evidence base, NOGG supported the use of calcium supplementation.^70^, ^71^ There is ample evidence to suggest that adequate protein intake is important for maintaining bone health in older adults.^72^, ^73^, ^74^ Given the high prevalence of malnutrition in PwP,^75^, ^76^ coupled with known competition for absorption between levodopa and dietary protein,^77^ particular consideration should be given to optimizing protein intake (eg, 1.0–1.2 g protein per kilogram of body weight per day) to maintain musculoskeletal health.^78^ Crucially, it is notable that all included guidelines were exclusively from high‐income European countries, and as such recommendations regarding diet in the prevention of osteoporosis may not be fully transferable to other countries with varying calcium intake, different dietary patterns, or different cultural practices relating to skin exposure.

### Physical Activity

Whereas 3 guidelines^42^, ^43^, ^46^ explicitly mentioned physical activity or exercise, limited additional guidance was provided as to what types of activity may be used to maintain and improve musculoskeletal health in PD. Many studies found a positive association between different exercise types in PD on both motor and nonmotor symptoms, including resistance exercise interventions.^79^, ^80^, ^81^, ^82^ Unfortunately, the impact of these interventions on bone health has not been explored, but it is plausible that improvements in BMD may occur. Given the high acceptability of exercise interventions among PwP^83^ and the success of programs such as PD‐Warrior^84^ and similar interventions,^85^ there may be appetite for musculoskeletal‐focused exercise regimens, which have been shown to be of benefit in non‐PD groups.^25^, ^26^ Bone‐targeted exercise programs and the wider adoption of PD‐focused specialized physiotherapy (eg, ParkinsonNet in the Netherlands)^86^ may both be acceptable and important avenues to maintain bone health and reduce fracture risk for PwP.

Although it was beyond the scope of the current project to derive an algorithm for clinical use in PD, Figure 2 provides a simplified set of recommendations that are readily adaptable to align with local best practice; however, this would require appropriate consumer input and consultation to generate actionable context‐specific guidance. It is recommended that a global working party be established to address this need and to provide overarching guidance that can be readily implemented and integrated with international best practice guidelines.

## Conclusion

Despite the high risk of fractures with associated morbidity and mortality in PwP, there is a paucity of clinical guidelines relating to bone health in this common and complex neurodegenerative condition. Routine screening of PwP for osteoporosis in clinical practice guidelines was not universally recommended despite its clinical importance, and specific treatment thresholds for PwP were lacking. There was inadequate advice regarding the prescribing of anti‐osteoporosis medications for PwP. Advice regarding modifiable lifestyle factors related to bone health such as nutrition and physical activity was not tailored to the specific needs of PwP. This review highlights the current lack of sufficient bone‐focused resources available internationally and the need to tailor such interventions for PwP that are specific to their country or cultural context.

## Author Roles

(1) Research project: A. Conception, B. Organization, C. Execution; (2) Statistical analysis: A. Design, B. Execution, C. Review and critique; (3) Manuscript preparation: A. Writing of the first draft, B. Review and critique.

M.Ó.B.: 1A, 1B, 1C, 3A, 3B

K.P.S.: 1C, 3B

F.E.L.: 1C, 3B

S.‐E.S.: 1C, 3B

J.M.: 3B

M.E.M.: 3B

V.M.: 3B

P.R.E.: 3B

J.Z.: 1C, 3B

A.Z.: 1A, 1C, 3A, 3B

## Disclosures

**Ethical Compliance Statement:** The authors confirm that the approval of an institutional review board was not required for this work. Informed patient consent was not necessary for this work. We confirm that we have read the journal's position on issues involved in ethical publication and affirm that this work is consistent with those guidelines.

**Funding Sources and Conflicts of Interest:** No specific funding was received for this work. The authors declare that there are no conflicts of interest relevant to this work.

**Financial Disclosures for the Previous 12 Months:** M.Ó.B., K.P.S., S.‐E.S., J.M., M.E.M., and V.M. declare that they have no financial disclosures to report. A.Z. has received funding from the National Centre for Healthy Ageing. F.E.L. is funded by the University of Auckland and High Value Nutrition National Science Challenge, New Zealand. P.R.E. has received grant funding to his institution from Amgen, Eli‐Lilly, and Alexion, and honoraria from Amgen, Kyowa Kirin, and Sanofi. J.Z. has received funding from the Medical Research Futures Fund (MRF2023806) and is an Australian Government Research Training Placement recipient.

## Supporting information


**Table S1.** Searches in MEDLINE, Embase, and Emcare databases.
**Table S2.** Searches in Web of Science database.

## Data Availability

Data sharing is not applicable to this article as no new data were created or analyzed in this study.
