# Supplementary material for: Management of Osteoporosis in Parkinson's Disease: A Systematic Review of Clinical Practice Guidelines
Source: Mov Disord Clin Pract. 2024 Dec 20;12(3):285–95. doi: 10.1002/mdc3.14311 (PMC11952945; doi:10.1002/mdc3.14311)
Supplement: Supplementary file 1 — Table S1. Searches in MEDLINE, Embase, and Emcare databases. Table S2. Searches in Web of Science database. [file MDC3-12-285-s001.docx]

Supplementary material

| Table S1. Searches in MEDLINE, Embase, and Emcare databases | | | | |
| --- | --- | --- | --- | --- |
| # | Term | MEDLINE | Embase | Emcare |
| 1 | Exp Parkinson disease/ | 85479 | 203964 | 23103 |
| 2 | (Parkinson or Parkinson*).mp | 164732 | 276089 | 44587 |
| 3 | 1 or 2 | 164732 | 276089 | 44587 |
| 4 | ((guidelin* or recommendation* or algorithm*) adj3 clinic*).mp | 73373 | 107256 | 37615 |
| 5 | ((consensus or position) adj2 statement) .mp | 12427 | 16259 | 5756 |
| 6 | 4 or 5 | 85129 | 122664 | 43071 |
| 7 | 3 and 6 | 359 | 812 | 208 |
| 8 | (osteop* or bone or fracture*).mp | 1333217 | 2140759 | 446349 |
| 9 | 7 and 8 | 8 | 48 | 14 |
|  | | | | |

| Table S2. Searches in Web of Science database | | |
| --- | --- | --- |
| # | Term | WoS |
| 1 | TS=(Parkinson OR Parkinson*) and Preprint Citation Index (Exclude – Database) | 360,444 |
| 2 | TS=((guidelin* OR recommendation* OR algorithm*) NEAR/3 clinic*) and Preprint Citation Index (Exclude – Database) | 131,218 |
| 3 | TS=((consensus OR position) NEAR/2 statement) and Preprint Citation Index (Exclude – Database) | 36,313 |
| 4 | #2 OR #3 and Preprint Citation Index (Exclude – Database) | 165,319 |
| 5 | #4 AND #1 and Preprint Citation Index (Exclude – Database) | 1,228 |
| 6 | TS=(osteop* OR bone OR fracture*) and Preprint Citation Index (Exclude – Database) | 4,672,525 |
| 7 | #6 AND #5 and Preprint Citation Index (Exclude – Database) | 44 |
|  | | |
